# Evaluation of the Highly Variable Agomelatine Pharmacokinetics in Chinese Healthy Subjects to Support Bioequivalence Study

**DOI:** 10.1371/journal.pone.0109300

**Published:** 2014-10-20

**Authors:** Qi Pei, Yan Wang, Zhe-Yi Hu, Shi-Kun Liu, Hong-Yi Tan, Cheng-Xian Guo, Ran-Ran Zhang, Yu-Xia Xiang, Jie Huang, Lu Huang, Hong Yuan, Guo-Ping Yang

**Affiliations:** 1 Center of Clinical Pharmacology, the Third Xiangya Hospital, Central South University, Changsha, Hunan, China; 2 Department of pharmacy, Changde First People's Hospital, Changde, Hunan, China; 3 Department of pharmacy, the Third Xiangya Hospital, Central South University, Changsha, Hunan, China; 4 Department of Clinical Pharmacy, College of Pharmacy, University of Tennessee Health Science Center, Memphis, Tennessee, United States of America; Penn State College of Medicine, United States of America

## Abstract

**Objectives:**

We aim to obtain the intra-subject coefficient of variability of a highly variable antidepressant agomelatine in humans, and propose an adjusted bioequivalence assessment strategy.

**Methods:**

A single-dose, randomized crossover design was conducted in four periods (reference administered thrice, placebo administered once) separated by seven days. A validated LC-MS/MS assay was used to measure drug concentrations in serial blood samples.

**Results:**

The intra-subject coefficient of variability was calculated using the residual variance of ANOVA analysis, and the results for C_max_ and AUC_0-t_ was 78.34% and 43.52%, respectively, in Chinese healthy subjects. The sample size required for standard BE study were 124(192, 340) if the expected deviation between the reference and generic products was set to 0 (5%, 10%).

**Conclusions:**

Agomelatine meets the criteria for highly variable drug in Chinese healthy male subjects, and the traditional BE criteria for agomelatine needs to be adjusted to alleviate the resource and ethical burden of using a large numbers of subjects in clinical trials. Our clinical data on the intra-subject variability of agomelatine PK in Chinese healthy population enables to adjust bioequivalence (BE) assessment approach for agomelatine based on the RSABE approaches recommended by regulatory agencies.

**Trial Registration:**

ChiCTR.org ChiCTR-TTRCC-13003835

## Introduction

Agomelatine brings a new concept to the antidepressant treatment. Compared to the other antidepressant drugs (e.g. mianserin and mirtazapine), agomelatine owns its melatonin (MT_1_ and MT_2_) agonist properties and a serotonin 5-HT_2C_ antagonist effect simultaneously [1], [2]. The recommended oral administration dose is 25 mg once daily [3]. The existing data on agomelatine metabolism, bioavailability and pharmacokinetics in Caucasian indicate that the absorption of agomelatine is rapid with the median t_max_ 0.75–1.5 hours and almost complete with at least 80% intestinal absorption [4], [5]. However its absolute oral bioavailability is low, approximately 3–4%, with highly variable (estimated to 104%), which has been explained by its extensive first pass metabolism [3].

For new generic agomelatine products/formulation with clinical trial permission of China Food and Drug Administration (CFDA), bioequivalence (BE) evaluation is one of the pivotal clinical studies required in support of its marketing application. Due to the highly variable feature, a standard number of subjects (e.g., 18–24) may not be able to demonstrate the bioequivalence of the generic products and their corresponding reference products using a two-way crossover design. In order to obtain adequate statistical power to meet BE limits, the subjects number for BE study of highly variable drugs need to be calculated based on the intra-subject coefficient of variability (CV) of pharmacokinetic (PK) parameters (C_max_ and AUC) of reference products [6], [7], [8]. To our knowledge, the intra-subject CV data of agomelatine in Chinese healthy volunteers has not been reported. So we aim to investigate the intra-subject CV of agomelatine in Chinese healthy volunteers, and propose an adjusted BE assessment strategy for agomelatine.

## Methods

The protocol for this trial and supporting CONSORT checklist are available as supporting information; see Checklist S1 and Protocol S1.

### Ethics Statement

The study was approved by the Ethics Committee of the Third Xiangya Hospital of Central South University, Changsha, Hunan P. R. China (approved number: 12066), on September 7, 2012, name: The intra-subject coefficient of variability of agomelatine in healthy Chinese and registered in the Chinese Clinical Trial Registry (registration number: ChiCTR-TTRCC-13003835). All clinical investigation must have been conducted according to the principles expressed in the Declaration of Helsinki. Written informed consents were obtained from all subjects before enrollment and overall clinical trial procedures abided by the Good Clinical Practice of the International Conference on Harmonization (ICH-GCP).

### Subjects

Suitable subjects were healthy males between 18 and 40 years of age with a Body Mass Index (BMI) between 19 and 25 kg·m^−2^. Subjects were judged to be eligible for the study when no clinical significant abnormal findings existed on a complete medical examination consisting of medical history, physical examination, 12-lead electrocardiogram, hematology, blood biochemistry and urinalysis.

### Study Design

This was a single-blind, two-treatment, four-period, four-sequence crossover design study (R-R-R-P, R-R-P-R, R-P-R-R, P-R-R-R). After 10-hour overnight fast in the ward, all subjects (n = 16) were randomly assigned to four groups and received a single oral 25 mg of agomelatine (reference, Valdoxan, Servier, Frence) in three of the four treatment periods and placebo in the other treatment period. Each study period was separated by 7 days. The placebo group was set to conduct safety evaluation.

Each dose was administrated with 250 ml of tap water. Light diets were served 4 and 10 hours after taking the dose. The same standardized diets were provided in all four periods. During each study day, no food except the standardized meals served was permitted. Neither caffeine-containing nor alcohol beverages were allowed until 24 hours after dosing. Smoking was forbidden during the same interval after the dose administration. Neither prescription nor over-the-counter drugs were allowed during participation in the study. No water drinking was required within 2 hours before and after the dose administration.

Serial blood samples, using anticoagulant tube were collected at 0, 0.25, 0.5, 0.75, 1.0, 1.5, 2.0, 2.5, 3.0, 4.0, 5.0, 6.0, 8.0, 10.0, 12.0 and 16.0 hours after dosing. The blood samples were centrifuged and the plasma samples were stored at −20°C until analyzed.

Safety was evaluated through the assessment of adverse events (AEs), vital signs and standard laboratory evaluation.

### Analytical Assay

An Agilent 1260 HPLC system (Agilent Technologies, Germany) was used to analyze the plasma samples. Mass spectrometric detection was performed on an Agilent 6460 triple quadrupolemass spectrometry (Agilent Technologies, Germany) using multiple reaction monitor (MRM). Data processing was performed on Agilent MassHunter Workstation Software (Agilent Technologies, German).

The HPLC separation was performed on an Agilent Phenomenex C_18_ column (4.6×150 mm, 5 µm) (Phenomenex, USA) by a mobile phase consisting of 5 mM ammonium formate (80∶20, v/v) at 35°C, and was delivered at a flow rate of 0.8 mL/min.

The typical operating source conditions for MS scan in positive ESI mode were optimized such that the capillary voltage was 4.0 kV and the skimmer at, 60 V. Nitrogen was used as the drying (350°C; 11 L/min) and nebulizer (45 psi) gas.

The multiple reaction monitoring (MRM) conditions were m/z 244→185.1 and m/z 386.1→122.0 for analyte and IS, respectively. The detetion parameters were optimized as follows: Collision energy, 10 eV for analyte and 21 eV for the IS; ionization voltage, 80 V for analyte and 95 V for the IS.

The analytical assay involved the addition of a 30 µL phenacetin (internal standard, IS) and 300 µL of plasma, followed by extraction into 4 mL of extraction solvent, ethyl acetate. The sample was centrifuged for 5 minutes and the organic phase was transferred into a polypropylene tube and evaporated to dryness at 45°C under a gentle stream of nitrogen. The residue was reconstituted in 200 µL of mobile phase, after high speed centrifugation, 3 µL was injected onto the LC-MS/MS system.

The response was linear in the concentration range, 0.04096 to 10 µg·L^−1^, with a coefficient of determination (r^2^)>0.997. The limit of quantitation was 0.04096 µg·L^−1^ and the inter-assay coefficient of variation at this concentration was 6.4%. The intra-assay and inter-assay precision of the quality control samples were <10%; the accuracy was within 15% of the nominal concentration. The methods were also validated for selectivity, carry-over effect, matrix effects, extraction recoveries and stability. All the validation results meet the accepted criteria according to the EMA guidance on bioanalytical method validation [9].

### Data Analysis

From the measured plasma concentration data, the area under the plasma concentration *vs*. time curve from zero to the last measurable time point (AUC_0-t_) was calculated by the linear trapezoidal method. The maximum plasma concentration (C_max_) was obtained directly from the plasma concentration-time curve. Analysis of variance (ANOVA) [10], using period, sequence as fixed model effects, and subject nested within sequence as random effects, were performed on the natural logarithmic transformations of C_max_ and AUC for the 3 same separated treatment periods using the GLM (General Linear Models) procedure of SPSS (Version 18.0, Chicago, IL, USA). Based on the literature [8], [11], [12], the mean square error (MSE) was used to estimate the intra-subject variation. The intra-individual CV was related to the MSE on the logarithmic scale as follows:
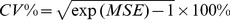
(1)


Sample sizes for the agomelatine BE study were calculated using the following formula [13]:
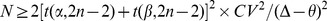
(2)where △ is indicative of BE limit, according to the current CFDA guidelines, usually set to ±20% for AUC and ±25% [14] for C_max_ in most BE studies; θ = [(µ_T_–µ_R_)/µ_R_]*100 is the difference in average bioavailability between the test and reference formulations; N is the total number of subjects required to achieve a 1-β(0.8 if β = 0.2) power at the α(e.g. 0.05) significance level.

## Results

### Demographics and safety

Sixteen healthy male volunteers (fifteen Han nationality and one Tujia minority), age 19–29 years (mean, 23 years), height 1.62–1.75 m (mean, 1.68 m), weight 50.5–68 kg (mean, 57 kg), BMI 19.1–22.2 kg·m^−2^ (mean, 20.3 kg·m^−2^) participated in the study.

There were no protocol violations or serious AEs observed during the study. Nine of the 16 subjects experienced a total of 11 AEs (9 were mild and 2 were moderate). The common types of AEs were somnolence (2 events) and tiredness (2 events), followed by the foot heel injury (1 event), amygdalitis (1 event), vasovagal reaction (1 event), globulin content decreased (1 event), aspartate aminotransferase and alanine aminotransferase increased (1 event), urticaria papulosa (1 event), rash (1 event). Several AEs (4 events) were considered to be probably related to the study medication. No clinically significant abnormalities on vital sign measurements or electrocardiographic recordings were reported. There was no significant difference in safety between the placebo group and experimental group.

### Pharmacokinetic parameters

Main pharmacokinetic (PK) parameters of agomelatine(25 mg oral) in 16 Chinese healthy male volunteers summarized in Table 1, where the data in Caucasian is provided for comparision [3]. There are significant ethnic differences between Chinese and Caucasian in the rate of absorption (C_max_) and the extent of absorption (AUC_0-t_) while no ethnic difference in t_max_ and t_1/2_. Less obvious first-pass effect in Chinese may partially account for why both C_max_ and AUC of Chinese healthy male were much higher than those of the Caucasian. C_max_ and AUC_(0-t)_ in each healthy Chinese volunteer of the three same separated treatment following oral agomelatine reference tablets were presented in Table 2.

**Table 1:** Comparison of the pharmacokinetic parameters of agomelatine following oral administration of 25 mg on three separate occasions in Chinese healthy subjects (n = 16).

Race	C_max_(ng/ml)^1^	AUC_0-t_ (ng/ml×h)^a^	t_max_ (h)^b^	t_1/2_ (h)^a^
Chinese(n = 16)	9.3±15.9	11.1±14.3	1 (0.5–4)	0.96±0.45
westerner(n = 8)[^3^]	3.0±2.8	4.9±5.6	1 (0.5–4)	0.9±0.4

a mean±SD;

b median (min-max).

**Table 2:** C_max_ and AUC_(0-t)_ of the three same separated treatment in each healthy Chinese volunteer following oral administration of 25 mg of agomelatine reference tablets (n = 16).

Subjects	C_max_(ng/mL)						AUC_(0-t)_ (ng/mL×h)					
	R_1_	R_2_	R_3_	Mean	SD	CV (%)	R_1_	R_2_	R_3_	Mean	SD	CV (%)
1	0.40	1.51	0.74	0.88	0.57	64.26	0.61	1.90	1.14	1.22	0.65	53.29
2	0.48	0.20	0.59	0.42	0.20	47.92	0.84	0.32	0.73	0.63	0.27	42.98
3	1.55	2.08	2.68	2.10	0.56	26.86	4.55	3.77	6.86	5.06	1.61	31.74
4	3.82	4.88	4.01	4.23	0.56	13.34	7.95	8.15	4.73	6.94	1.92	27.59
5	19.02	25.91	27.86	24.26	4.64	19.14	27.82	26.28	37.93	30.68	6.33	20.63
6	1.77	8.41	2.18	4.12	3.72	90.27	4.87	7.81	3.18	5.29	2.34	44.24
7	3.48	2.94	9.51	5.31	3.64	68.67	9.16	8.58	10.74	9.49	1.12	11.77
8	2.59	0.54	0.87	1.34	1.10	82.57	1.98	1.34	1.50	1.61	0.33	20.69
9	7.72	19.07	9.28	12.02	6.15	51.17	14.69	18.73	12.28	15.23	3.25	21.36
10	8.70	4.45	6.92	6.69	2.13	31.91	10.79	7.33	7.80	8.64	1.88	21.77
11	5.25	12.63	11.68	9.85	4.01	40.72	9.73	12.69	10.48	10.97	1.54	14.02
12	9.71	4.53	11.63	8.63	3.67	42.57	15.15	9.81	12.58	12.51	2.67	21.32
13	18.93	5.85	1.78	8.85	8.96	101.27	14.03	7.10	4.11	8.42	5.09	60.47
14	51.84	14.53	96.92	54.43	41.26	75.80	53.80	31.31	77.13	54.08	22.91	42.37
15	2.44	1.49	0.48	1.47	0.98	66.77	2.34	3.67	1.65	2.56	1.02	40.07
16	8.72	4.27	0.95	4.65	3.90	83.89	7.17	4.58	1.40	4.38	2.89	65.87

### Concentration-time curves

**Figure 1:**
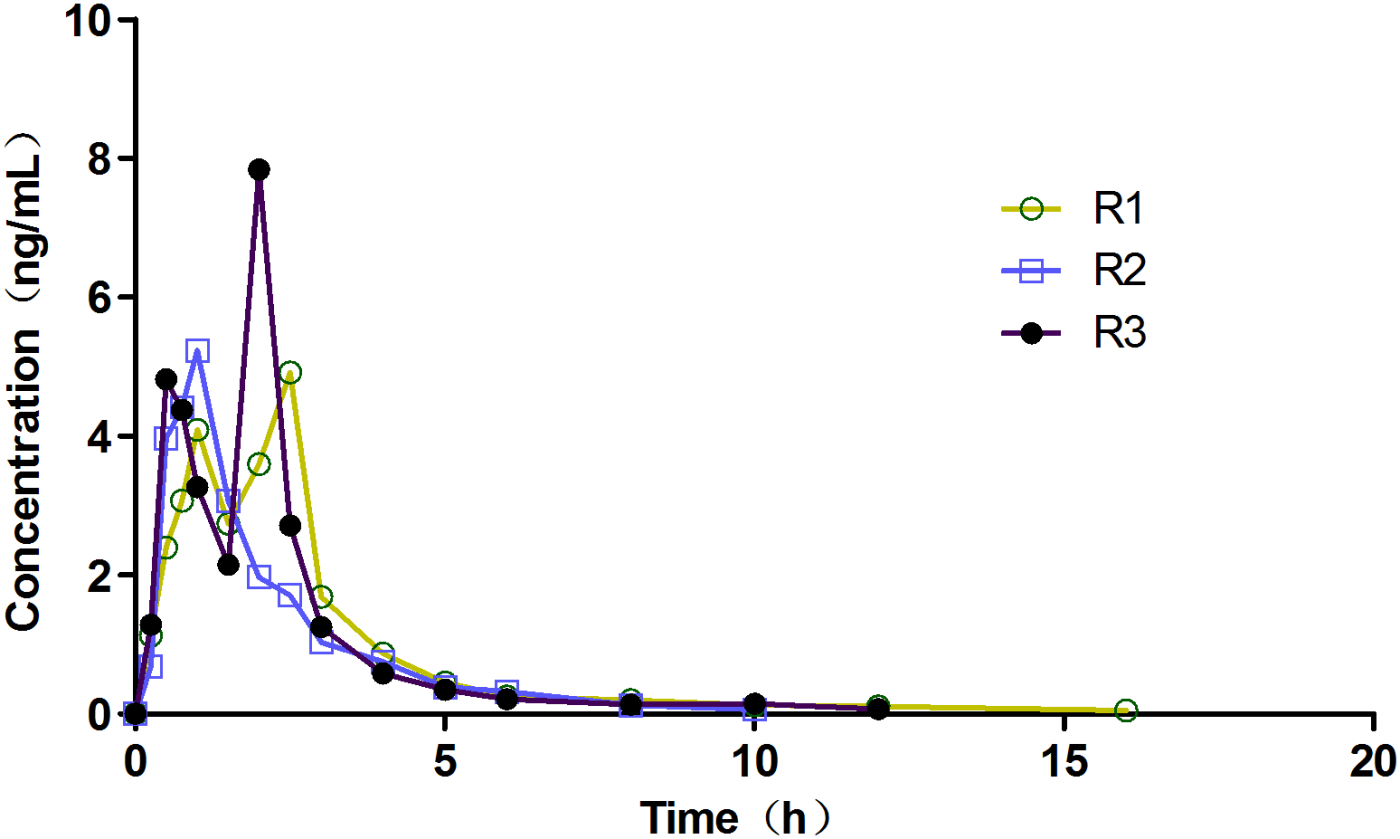
Mean plasma agomelatine concentration–time profiles following oral administration of 25 mg of agomelatine reference tablets in healthy Chinese volunteers (n = 16). R1, R2 and R3 represented the three same separated treatments, respectively.

Mean plasma concentration-time profiles of agomelatine from 16 Chinese subjects after a single oral dose of 25 mg agomelatine reference tablet were illustrated in Figure 1. The concentration-time profiles differed greatly in three periods. Particularly, double-peak phenomenon was observed for two of the profiles (R1 and R3), indicating notable intra-individual variability.

### Intra-subject variability

**Figure 2:**
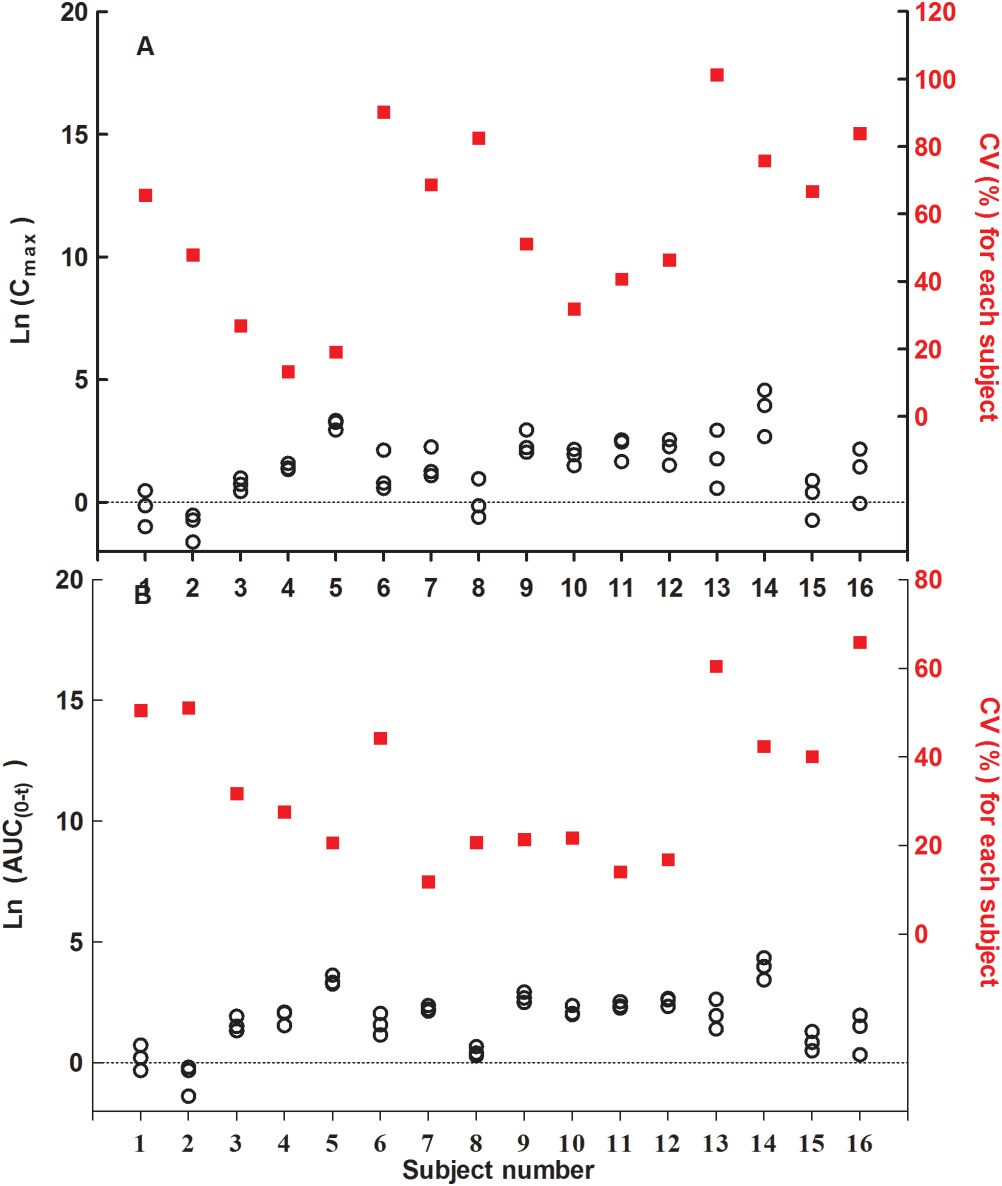
C_max_(A) and AUC_0-t_ (B) values after natural logarithmic transformation and intra-subject CV(the ratio of the standard deviations to mean) (n = 16).

The C_max_ and AUC_0-t_ values after natural logarithmic transformation and intra-subject CV (the ration of the standard deviations to mean) of all 16 subjects on each occasion were shown in Figure 2. The overall intra-subject CV was calculated by taking the square root of the MSE on the logarithmic scale and multiplying by 100% as [eq.1]. There were notable intra-subject variabilities in AUC_0-t_ (CV = 43.52%) and C_max_ (CV = 78.34%).

As the absolute values of AUC_0-t_ or C_max_ for some subjects (e.g., subject 1, 2, 8 and 15) in different periods are close to each other, especially due to low values the relative CV (the ratio of standard deviation to mean) was used in Figure 2. The intra-subject CV of C_max_ varied from 13.34% to 101.27% (average 57.02%, 14 of 16 subjects >30%) and the AUC_0-t_ varied from 11.77% to 65.87% (average 33.82%, 8 of 16 subjects >30%).

### Sample size calculation for standard BE study

Table 3 presents the required total sample size to achieve an 80% power for θ from 0 to 10% by increment of 5%. If expected deviation was 0 (5%, 10%) between the reference and generic products, the sample size required by standard BE study was 124 (192, 340).

**Table 3:** Sample size calculation for standard agomelatine bioequivalence study in Chinese healthy volunteers.

PK Parameters	α	1-β	CV (%)	△(%)	θ (%)	N
AUC_0-t_	0.05	0.80	43.52	0.20	0	62
					5	108
					10	242
C_max_	0.05	0.80	78.34	0.25	0	124
					5	192
					10	340

### Sample size calculation for reference-scaled average bioequivalence (RSABE)

In this study, C_max_ was chosen to be the basis for the sample size calculation for the higher variability than AUC. Table 4 shows the sample sizes required for the design of agomelatine BE studies using RSABE approaches. The ratios of geometric means (GMR) are considered within the range of 0.90 to 1.00. Based on the EMA approach, the minimum number of subjects for the 3-period partially replicating design is 72, 54 and 51 for GMR 0.90, 0.95 and 1.00, respectively. Using the similar approach, the number of subjects needed for the 4-period replicated design is 50, 38 and 35, respectively. Based on the FDA approach, the minimum number of subjects for the 3-period partially replicating design is 40, 29 and 29 for GMR 0.90, 0.95 and 1.00, respectively. For the 4-period replicated design, the number of subjects is 29, 21 and 20, respectively. Haidar et al [15] suggested that the inclusion of at least 24 subjects were required for Caucasian BE study. So, the same 24 subjects could be considered as the absolute minimum in Chinese BE study.

**Table 4:** Sample sizes enquired for BE study of agomelatine using EMA and FDA approaches.

Regulatory Agencies	Design	Ratio of geometric means (GMR)		
		0.90	0.95	1.00
EMA	3-period study	72^a^	54	51
	4-period study	50	38	35
FDA	3-period study	40	29	27
	4-period study	29	21	20

a Number of human subjects.

## Discussion

This study reported the intra-subject CV data of agomelatine pharmacokinetics in Chinese healthy male subjects (within-subject variability is >30%) for the first time, demonstrating agomelatine meets the criteria for highly variable drug in Chinese subjects. Traditional BE assessment approach needs to be adjusted for highly variable drugs based on essential pilot data. Although agomelatine pilot data is available in literature for Caucasians [3], it cannot be directly applied to the BE study in other populations due to ethnic differences, which was demonstrated in the pharmacokinetic parameters of agomelatine by comparing the current Chinese study to the Caucasian study.

For highly variable drug of agomelatine, too large sample size required by standard BE study. Therefore, adjustment of the traditional BE criteria and study design is needed to alleviate the resource and ethical burden of using a large number of subjects in clinical trials. To lower sample size required for highly variable drugs BE study, the regulatory agencies, Food and Drug Administration (FDA) and European Medicines Agency (EMA), have recommended the RSABE approach, whereby the BE acceptance limits are scaled to the variability of the reference product [7], [15], [16].

FDA has posted Guidance for Industry (Bioequivalence Recommendations for Progesterone Oral Capsule [17], Warfarin Sodium [18]) which providing step-by-step instructions on how to analyze data using RSABE. Meanwhile, Tothfalusi L [19] conducted massive simulations, from which sample sizes needed for BE assessment of highly variable drugs with different with-subject variable were calculated by using the RSABE approaches of FDA and EMA. It presented the detailed sample size in four tables based on different power (80%, 90%) and intra-subject CV (30%∼80%).

Therefore, our work is a pilot study to estimate the BE study sample size to facilitate generic application based on the new RSABE approach, with significance of providing intra-subject CV in Chinese healthy male.

Intra-subject CV was calculated based on the formula CV = [exp (MSE)-1]^1/2^ and related to the MSE obtained by the GLM procedure of SPSS in this study. Several studies [10], [11] about intra-subject variance (σ_i_^2^) were similar to our work, such as in the assumption of model for a standard 2×2 crossover design, that the σ_i_^2^ was indicative of the residual variance and calculated by using ANOVA. Unlike the 3 same separated treatments of this work, the fixed model effects of the classical 2×2 crossover included formulation. In the FDA guidance on progesterone BE, the formula of CV_WR_ = [exp(s^2^_WR_)-1]^1/2^ was used to calculate the intra-subject CV. s^2^_WR_ is the population within-subject variance of the reference formulation. The guidance recommends researchers to adopt PROC GLM and MIXED of SAS for 3-way and 4-way replicated BE studies, respectively [17].

A number of factors can contribute to the high inter-individual variability in agomelatine pharmacokinetics. Agomelatine undergoes an extensive first pass metabolism after oral administration resulting in remarkable low systemic availability. CYP1A2 is the major enzyme (90%) involved in the hepatic metabolism of agomelatine, and smoking was shown to reduce plasma concentrations three- to fourfold due to induction of CYP1A2. Subjects with smoking habit would contribute to the high variability in exposures of agomelatine. Furthermore, oral administrated agomelatine is extensively distributed throughout the body which could be another factor contributing to high inter-individual variability [3], [20].

Recently, Song [21] found that several CYP1A2 SNPs (rs762551, rs2470890 and rs2472304) might be associated with the marked interindividual variability of agomelatine. Whether these polymorphisms affect the intra-subject variability of agomelatine PK in Chinese healthy male was not considered in our study, which was a limitation in this article.

## Conclusions

Agomelatine meets the criteria for highly variable drug in Chinese subjects, and the traditional BE criteria for agomelatine needs to be adjusted to alleviate the resource and ethical burden of using a large numbers of subjects in clinical trials. Our clinical data on the intra-subject variability of agomelatine PK in Chinese healthy population recommends adjusting bioequivalence (BE) assessment approach for agomelatine based on the RSABE approaches recommended by regulatory agencies.

## Supporting Information

Figure S1 **Agomelatine CONSORT Flow Diagram.**
(TIFF)

Checklist S1 **CONSORT checklist.**
(DOCX)

Protocol S1 **Study on the Variability of Agomelatine.**
(DOC)

Protocol S2 **Chinese Version protocol.**
(DOC)
