# Supplementary figures and images for: Evaluation of the Highly Variable Agomelatine Pharmacokinetics in Chinese Healthy Subjects to Support Bioequivalence Study
Source: PLoS One. 2014 Oct 20;9(10):e109300. doi: 10.1371/journal.pone.0109300 (PMC4203722; doi:10.1371/journal.pone.0109300)

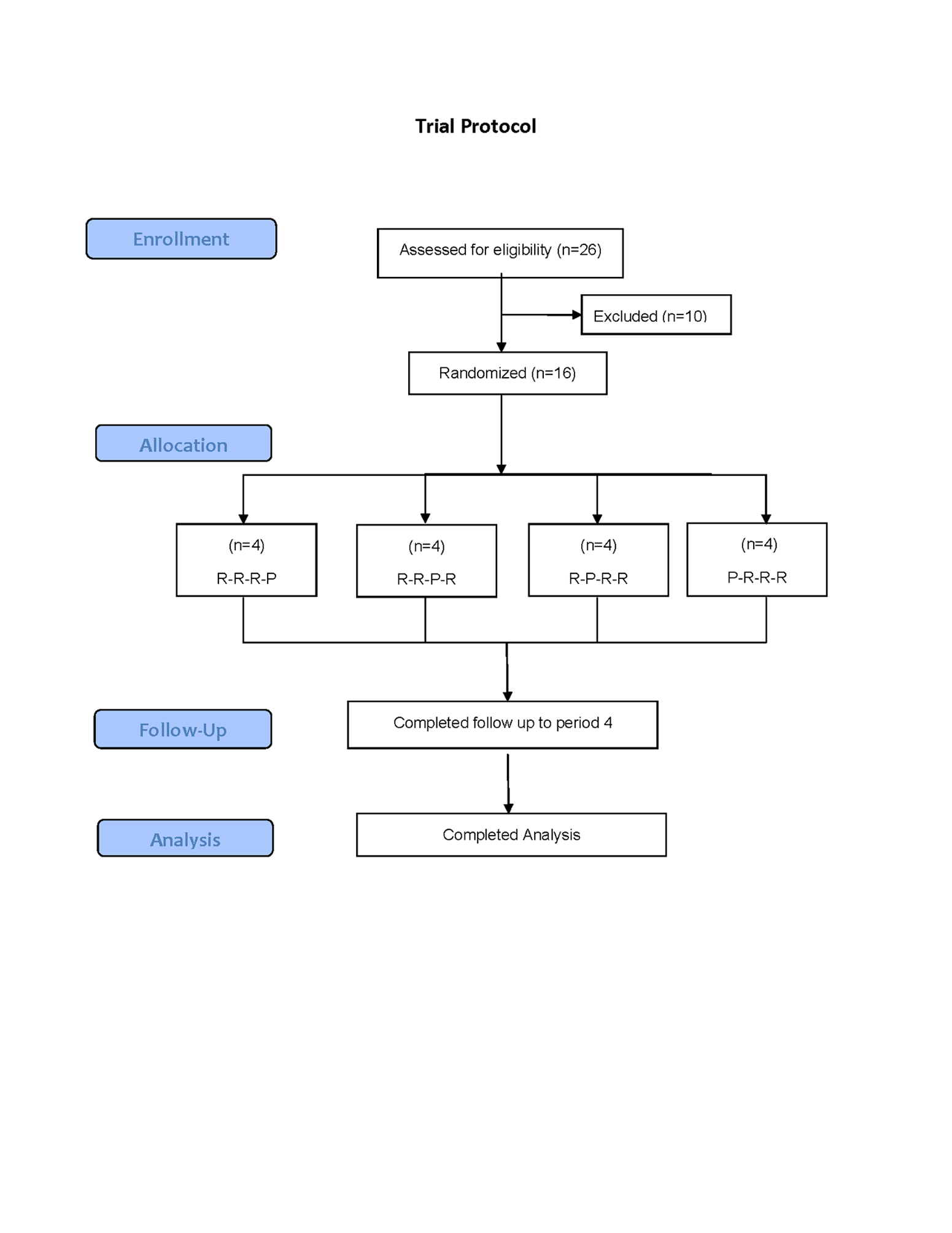

Supplement: Figure S1 — Agomelatine CONSORT Flow Diagram. (TIFF) [file pone.0109300.s001.tiff]
