# Supplementary material for: Evaluation of the Highly Variable Agomelatine Pharmacokinetics in Chinese Healthy Subjects to Support Bioequivalence Study
Source: PLoS One. 2014 Oct 20;9(10):e109300. doi: 10.1371/journal.pone.0109300 (PMC4203722; doi:10.1371/journal.pone.0109300)
Supplement: Protocol S1 — Study on the Variability of Agomelatine. (DOC) [file pone.0109300.s003.doc]

|  | | |
| --- | --- | --- |
| Clinical Investigation Protocol | | |
| **Study on the Variability of Agomelatine** | | |
|  | | |
| Clinical Trial Authorization NO. 2012L01023 | | |
| Protocol NO.XY3-AGO1208A01 | | |
|  | |  |
| Principal Investigator: | Guoping Yang | |
| Protocol Designer: | Guoping Yang | |
| Leading Researchers: | Guoping Yang, Qi Pei, Yan Wang, Ranran Zhang, Lu Huang, Hongyi Tan, Chengxian Guo, Zhe Yi Hu, Yu Xia Xiang, Hong Yuan | |
| - [Research](app:ds:research) [Institution](app:ds:institution): | the Third Xiangya Hospital, Central South University  Address, 138 Tong Zi Po Road, YueLu District, Changsha, Hunan, China  Telephone, +86 0731-88618339  Postcode 410013 | |
| version number： | 1.0 | |
| Version Date： | August 28, 2012 | |

**Catalogues**

[**Ethical Approval** 4](#__RefHeading___Toc397124069)

[**Trial Protocol Abstract** 6](#__RefHeading___Toc397124070)

[**缩略语对照表** 12](#__RefHeading___Toc397124071)

[1. 研究背景 14](#__RefHeading___Toc397124072)

[1.1．药理毒理 14](#__RefHeading___Toc397124073)

[1.2．药代动力学 15](#__RefHeading___Toc397124074)

[1.3．不良反应和副作用 15](#__RefHeading___Toc397124075)

[1.4．临床应用 15](#__RefHeading___Toc397124076)

[2．试验目的 15](#__RefHeading___Toc397124077)

[3．研究方案依据 15](#__RefHeading___Toc397124079)

[4．试验负责人及主要研究人员简介 16](#__RefHeading___Toc397124080)

[5．试验用药 16](#__RefHeading___Toc397124081)

[6．受试者选择 16](#__RefHeading___Toc397124082)

[6.1．筛选步骤 16](#__RefHeading___Toc397124083)

[6.2． 入选标准 17](#__RefHeading___Toc397124084)

[6.3．排除标准 17](#__RefHeading___Toc397124085)

[6.4． 剔除标准： 18](#__RefHeading___Toc397124086)

[7. 剂量及确定依据 19](#__RefHeading___Toc397124087)

[8. 试验设计及方案描述 19](#__RefHeading___Toc397124088)

[8.1．受试者例数 19](#__RefHeading___Toc397124089)

[8.2．给药途径和给药方案 19](#__RefHeading___Toc397124090)

[8.3试验设计 19](#__RefHeading___Toc397124091)

[8.4．试验方法 21](#__RefHeading___Toc397124092)

[8.5.试验前后体检及试验期间随访观察 22](#__RefHeading___Toc397124093)

[8.6． 生物样品分析方法 22](#__RefHeading___Toc397124094)

[9．数据处理与统计 22](#__RefHeading___Toc397124095)

[9.1. 数据提供 22](#__RefHeading___Toc397124096)

[9.2．药动学参数估算方法 22](#__RefHeading___Toc397124097)

[10．统计分析 23](#__RefHeading___Toc397124098)

[11. 安全性评价 23](#__RefHeading___Toc397124099)

[11.1 医疗监护 23](#__RefHeading___Toc397124100)

[11.2不良事件 24](#__RefHeading___Toc397124101)

[11.3．严重不良事件 25](#__RefHeading___Toc397124102)

[11.4．阿戈美拉汀片可能出现的不良反应及抢救措施 26](#__RefHeading___Toc397124103)

[11.5．终止试验标准 26](#__RefHeading___Toc397124104)

[12． 伦理要求 26](#__RefHeading___Toc397124105)

[13 . 试验管理与质量控制 27](#__RefHeading___Toc397124106)

[13.1．管理机构和实施GCP的情况 27](#__RefHeading___Toc397124107)

[13.2．各方职责 27](#__RefHeading___Toc397124108)

[13.3．方案修订 28](#__RefHeading___Toc397124109)

[13.4．受试者管理 28](#__RefHeading___Toc397124110)

[13.5．试验药物管理 28](#__RefHeading___Toc397124111)

[13.6．给药依从性管理 29](#__RefHeading___Toc397124112)

[13.7．受试者退出试验的管理 29](#__RefHeading___Toc397124113)

[13.8．伴随治疗 29](#__RefHeading___Toc397124114)

[13.9．数据记录与文件保留 29](#__RefHeading___Toc397124115)

[13.10．个人资料的保密 30](#__RefHeading___Toc397124116)

[13.11．质量控制 30](#__RefHeading___Toc397124117)

[14．参考文献 30](#__RefHeading___Toc397124118)

# **Ethical Approval**

**1. Documents should be adhered**

Clinical trials must be undertaken to the demands in the [standard](app:ds:standard) [operating](app:ds:operating) [procedure](app:ds:procedure) of Department of Clinical Study Phase I, the Third Xiangya Hospital of Central South University, and should be complied with the trial protocol which should be designed to follow the following documents:

1. [Declaration](app:ds:Declaration) [of](app:ds:of) [Helsinki](app:ds:Helsinki)
2. [The](app:ds:The) [Drug](app:ds:Drug) [Administration](app:ds:Administration) [Law](app:ds:Law) [of](app:ds:of) [the](app:ds:the) [PRC](app:ds:PRC)
3. Drug Registration Regulation
4. the Criterions for the Quality Control of Clinical Trial of Drugs
5. Chemical drug preparations of human bioavailability and bioequivalence study technical guidelines

**2.** **Institutional Review Board and the** [**Ethics**](app:ds:Ethics) [**Committee**](app:ds:Committee)

Before the clinical trial begin，the present trial protocol, [informed](app:ds:informed) [consent](app:ds:consent)，the approval documents of the relevant authorities, drug testing report and any type of subjects ecruitment advertisement should be submitted to the Institutional Review Board or other organization had equal rights by the investigator for approval.

**3. Informed Consent Form**

[Informed](app:ds:informed) [consent](app:ds:consent) form should be submitted to the relevant Ethics Committee for approval，all the related content of the trial protocol must be included .

The potential risks and hazards of the trial should be clearly explained to all the potential subjects before any activities related to the trial begining.

Informed consent form is may be signed and dated only after the subject fully understands the explanations on the nature and aim of the trial, the investigator needs to provide subjects a copy of the informed consent form.

# **Trial Protocol Abstract**

| **Basic information about clinical trials**  **Clinical Trial Authorization Number,** 2012L01023  **Protocol Number**, XY3-AGO1208A01  **Version Date**, August 28, 2012  **Investigational Drugs**，Agomelatine  [**Research**](app:ds:research) [**Institution**](app:ds:institution)，Department of Clinical Study Phase I ,the Third Xiangya Hospital, Central South University  **principal investigator**， Guoping Yang |
| --- |
| **Title**  Study on the Variability of Agomelatine |
| **Objective**  We aim to obtain the intra-subject coefficient of variability of a highly variable antidepressant agomelatine（Valdoxan, 25mg/ tablet, Servier，Frence） in The Chinese male volunteers, and propose an adjusted bioequivalence assessment strategy between reference tablets from Servier and test tablets from domestic companies. |
| **Trail design**  This was a single-blind, two-treatment, 4-period, 4-sequence crossover design study(R-R-R-P, R-R-P-R, R-P-R-R, P-R-R-R). After 10-hour overnight fast in the ward, a single oral 25 mg of agomelatine (reference, Valdoxan, Servier，Frence) in three of the four treatment periods and receive [placebo](javascript:showjdsw('showjd_0','j_0')) in the other treatment period were randomly assigned to all subjects (n = 16) receive. Each study period was separated by 7 days. The placebo group was setting to conduct safety evaluation. The plasma concentrations of Agomelatine of different time point were determined by HPLC-MS/MS.  **Subjects**  16 appropriated healthy male volunteers were selected to participate in this study (The inclusion criteria and exclusion criteria would be present below)  **Study Procedures**  **Screening of the subjects：**subjects were screened according to the inclusion criteria and exclusion criteria.  Grouping：4 groups were obtained after randomized according to the recruitment order, see randomized grouping.  **The first period：**After 10-hour overnight fast in the ward, a single oral 25 mg of agomelatine (reference, Valdoxan, Servier，Frence) on the next morning in three of the four treatment periods and receive [placebo](javascript:showjdsw('showjd_0','j_0')) in the other treatment period were randomly assigned to all subjects (n = 16) receive. Tablets were taken with 250ml warm water. No water drinking was required within 2 hours before and after the dose administration.Light diets were served 4 and 10 hours after taking the dose. Serial blood samples , using anticoagulant tube were collected at 0, 0.25, 0.5, 0.75,1.0, 1.5, 2.0, 2.5, 3.0, 4.0, 5.0, 6.0, 8.0, 10.0, 12.0 and 16.0 hours after dosing via elbow vein(5ml for every time point). To follow-up before and 2 hours,12 hours,24 hours after dosing, Safety was evaluated through assessment of adverse events (AEs), vital signs and standard laboratory evaluation. All drug combinations should be recorded.  [**Washout**](app:ds:washout) [**period**](app:ds:period)**：**Each study period was separated by 7 days。  **The second, third and fourth period:** randomly receive [placebo](javascript:showjdsw('showjd_0','j_0')) or agomelatine as the first period.  **The last posttreatment follow-up:** Subjects receive physical and medical examination 24 hours after the last dosing, continue to follow-up to the normal or stability If there are clinical significance of abnormal.  **Biological sample analysis**  The plasma concentrations of Agomelatine of different time point were determined by HPLC-MS/MS. The pharmacokinetics parameters were calculated according to the plasma concentration – time data and the actually sampling time.  **Pharmacokinetics parameters**  The plasma concentration-time curves of agomelatine fitted with the non-compartment model after single oral dose of agomelatine tablets, and the data were processed by WinNonlin software to calculate the pharmacokinetic parameters.  **Statistical method**  From the measured plasma concentration data, the area under the plasma concentration *vs*. time curve from zero to the last measurable time point (AUC_0-t_) was calculated by the linear trapezoidal method. The maximum plasma concentration (C_max_) was obtained directly from the plasma concentration-time curve. Analysis of variance (ANOVA), including period, sequence and subject nested within sequence, were performed on the natural logarithmic transformations of C_max_ and AUC for the 3 same separated treatment periods using the GLM (General Linear Models) procedure of SPSS (Version 18.0, Chicago, IL, USA). Assume that all the mean square error (MSE) was caused by the intra-subject variation. The intra-individual CV was related to the MSE on the logarithmic scale as follows:  $CV\%=\sqrt{\exp(MSE)-1}\times100\text{\%}$[eq.1]  **Safety evaluation**  Clinical monitoring would be required to conducted by experienced clinicians and more intensive clinical care and observation are needed for all kinds of reaction, the occurrence of adverse events, the occurrence frequency, disposal and prognosis should also be recorded. Clinicians Major indicators, include [vital](app:ds:vital) [signs](app:ds:signs) and laboratory parameters, should be taken into analysis. The relationship between significant adverse event and the study drug will be evaluated. |
| **Inclusion criteria**  All participants should not be recruited until meeting the following criteria   1. male； 2. 18~40 years，and gap between the same batch less than 10; 3. Weight is greater than 50 kg，body weight index between 19 to 24 kg/m^2^（BMI=weight（kg）/height ^2^（m^2^））；      1. Based on the [physical](app:ds:physical) [examination](app:ds:examination), medical history, vital signs, electrocardiogram (ECG) and clinical laboratory examination results, subjects who were considered as in good health condition by clinicians, should have no history of metabolic abnormalities, mental, digestive tract, nervous system, liver, kidney, disorder, etc. ECG, heart rate, blood pressure, respiratory condition and laboratory examination including blood routine, urine, various biochemical examination (hepatic and renal function) without [abnormalities](app:ds:abnormalities) or have no clinical significance (depends the clinicians’ jugdement) 2. No history of allergies，have not taking any other drugs within two weeks; 3. No smoking and drinking habits; 4. Well informed about the clinical trial and sign the consents voluntarily   **Exclusion criteria：**  Subjects with one or more of the following standards will be ruled out：   1. Allergic constitution (Allergic to two or more drugs, food or pollen), allergic to the known medicine components, or have a clear food allergy; 2. Subjects who have the history of orthostatic hypotension； 3. Subjects who have the history of Specific allergic reaction （such as asthma, rubella, eczema dermatitis）, arrhythmia, bronchus or cardiovascular disease, diabetes, thyroid function hyperfunction, Parkinson's disease, or any other disease or physiological condition that can affect the trial result; 4. Subjects who are subjecting or have subjected to disease of GI problems, spasm, peptic ulcer , urinary infarction, mechanical ileus, ureteral spasm, biliary ,depression, liver, or disease that can affect the absorption and metabolism of the study drug; 5. Sever hemorrhage factors that can affect the elbow vein sampling; 6. Subjects who have the history of drug abuse or positive result of urinary drug within five years; 7. Subjects who have [electrocardiographic](app:ds:electrocardiographic) [abnormality](app:ds:abnormality) (Clinical significance) or vital signs [abnormality](app:ds:abnormality) （[systolic](app:ds:systolic) [pressure](app:ds:pressure) <90 mmHg or >140 mmHg，[diastolic](app:ds:diastolic) [blood](app:ds:blood) [pressure](app:ds:pressure) <50 mmHg or >90 mmHg；[heart](app:ds:heart) [rate](app:ds:rate) <50 bpm or >100 bpm）; 8. Positive for HIV ,HbsAG or HCV test; 9. Frequent drinkers who regular drinking more than 14 units for alcohol a week (one unit of alcohol is equal to 360ml beer, 150ml of wine and 45ml of spirits containing 90% alcohol) within 6 months or during the clinical trial; 10. Subjects who have taken soft drugs（such as marihuana）within 3 months or on trial, someone who have taken hard drugs (such as cocaine benzene piperidine, etc)within one year or on trial process; 11. Subjects who have taken drugs that can induce or inhibit the drug liver metabolism（inducer agent, such as Barbitone, carbamazepine, phenytoin glucocorticoid, omeprazole, inhibitor such as SSRI antidepressants, cimetidine, diltiazem, large ring lactone, nitro imidazoles, sedative hypnotics, verapamil, fluoroquinolone, class antihistamines）within 30 days or during the trial; 12. Subjects who have taken any kind of drugs within 14 days; 13. Subjects who have taken participant to any other clinical trials within 30 days; 14. Subjects who have donated blood or being taken sample within 3 months; 15. [Blood](app:ds:blood) [coagulation](app:ds:coagulation) [disorders](app:ds:disorders) or subjects who have subjected to thromboembolism disease; 16. Daily smoking more than one within 3 months or subjects who take any kinds of tobacco product during the trial; 17. Subjects who have a food allergy or special dietary requirements that can’t accept the uniform diets; 18. Subjects who have a history of malignancy; 19. Subjects who have a history of migraine or headache;Subjects who drink too much tea or coffee or other kinds of beverage containing [caffeine](app:ds:caffeine) （more than 8 cups）; 20. Subjects who was considered not appropriate for the trial by the researcher. |
| **The specification and lot number of agomelatine**  Produced by LES LABORATOIRES SERVIER, agomelatine tablet, specification：25 mg; lot number：893158; administration route: oral; dose:25mg；period of validity：can be saved to July 2014； [storage](app:ds:storage) [condition](app:ds:condition)：be hermetic and save below 25℃。 |
| **Ethic**  The trial protocol must be submitted to the ethics committee for approval, and all screening subjects should sign a inform consent form before. Trial must be going strictly according to the design of protocol and the requirement of GCP, and fully protect the legitimate rights and interests of the subjects. |

# 缩略语对照表

| 短语和专门术语 | 解释 |
| --- | --- |
| AE | 不良事件 |
| ALT | 丙氨酸氨基转移酶 |
| ANOVA | 方差分析 |
| AST | 天门冬氨酸氨基转移酶 |
| AUC_0~t_ | 从零到最后可测浓度的血药浓度-时间曲线下面积 |
| AUC_0~∞_ | 从零到无穷大时间内血药浓度-时间曲线下面积 |
| BMI | 体重指数 |
| BP | 血压 |
| bpm | 每分钟心跳次数（心率） |
| C_max_ | 观察到的最高血药浓度 |
| CL/F | 表观清除率/ F |
| ECG | 心电图 |
| EC | 伦理委员会 |
| F | 生物利用度 |
| GCP | 药物临床试验质量管理规范 |
| HIV | 人免疫缺陷病毒 |
| K_e_ | 消除速率常数 |
| LLOQ | 定量下限 |
| Ln | 自然对数（等同于ln） |
| mmHg | 毫米汞柱 |
| rpm | 每分钟转数 |
| SAE | 严重不良事件 |
| SFDA | 国家食品药品监督管理局 |
| SOP | 标准操作规程 |
| T_max_ | 观察到的达峰值浓度的时间 |
| t_1/2_ | 血药浓度减少一半所需时间 |
| V_d_/F | 表观分布体积/ F |

**试验流程图**

| **试验程序** | **知情**  **同意** | **筛选**  **基线** | **第一**  **周期** | | **洗脱期** | **第二**  **周期** | | **洗脱期** | **第三**  **周期** | | **洗脱期** | **第四**  **周期** | **出组** |
| --- | --- | --- | --- | --- | --- | --- | --- | --- | --- | --- | --- | --- | --- |
| 时 间 | **启动会~-1天** | **-7~-1天** | **1天** | **2天** | **3-7天** | **8天** | **9天** | **10-14天** | **15天** | **16天** | **17-21天** | **22天** | **23天** |
| 签署知情同意书 | X |  |  |  |  |  |  |  |  |  |  |  |  |
| 人口学资料 | X |  |  |  |  |  |  |  |  |  |  |  |  |
| 询问病史 | X |  |  |  |  |  |  |  |  |  |  |  |  |
| 近期和当前的用药史 | X |  |  |  |  |  |  |  |  |  |  |  |  |
| 体格检查 |  | X |  |  |  |  |  |  |  |  |  |  |  |
| 生命体征 |  | X | X | X |  | X | X |  | X | X |  | X | X |
| 心电图 |  | X |  |  |  |  |  |  |  |  |  |  | X |
| 血常规 |  | X |  |  |  |  |  |  |  |  |  |  | X |
| 尿常规 |  | X |  |  |  |  |  |  |  |  |  |  | X |
| 肝、肾功能 |  | X |  |  |  |  |  |  |  |  |  |  | X |
| 血糖 |  | X |  |  |  |  |  |  |  |  |  |  | X |
| 凝血功能 |  | X |  |  |  |  |  |  |  |  |  |  | X |
| 输血四项 |  | X |  |  |  |  |  |  |  |  |  |  |  |
| 随机 |  | X |  |  |  |  |  |  |  |  |  |  |  |
| 研究药物发放 |  |  | X |  |  | X |  |  | X |  |  | X |  |
| 血样采集 |  |  | X |  |  | X |  |  | X |  |  | X |  |
| 记录合并用药 |  |  | X | X | X | X | X | X | X | X | X | X | X |
| 不良事件记录 |  |  | X | X | X | X | X | X | X | X | X | X | X |

1. 生命体征测量：入住一期病房时、给药前及给药后2小时、12小时、24小时。
2. 心电图：标准的12导联ECG。
3. 血常规检查项目：白细胞计数、红细胞计数、血红蛋白、血小板计数、中性粒细胞百分比；

4）尿常规检查项目：蛋白质、葡萄糖、酮体、白细胞、红细胞、潜血、pH值；

5）**肝、肾功能检查项目：**丙氨酸氨基转移酶、天门冬氨酸氨基转移酶、总蛋白、白蛋白、总胆红素、直接胆红素、肌酐、尿素氮、尿酸;

6｝凝血功能检查项目：凝血酶原时间、活化部分凝血活酶时间；

7）输血四项检查项目：乙肝表面抗原（ HbsAg）、丙肝病毒抗体（HCV）、梅毒螺旋体抗体（RPR）、艾滋病病毒抗体(HIV)

**研究方案正文**

## 1. 研究背景

阿戈美拉汀（agomelatine）是褪黑激素（melatonin, MT）受体激动剂，同时也是5-羟色胺(5-HT) 2C 受体拮抗剂。是法国施维雅公司研发的第一个褪黑激素类抗抑郁药, 能有效治疗抑郁症, 尤其对重度抑郁症疗效明显, 并能有效改善睡眠参数和保持性功能，不良反应小。阿戈美拉汀的化学名称：N-[2-(7-甲氧基-1-萘基)乙基]乙酰胺。分子式：C15H17NO2，分子量：243.3。化学结构如下图所示：

## 1.1．药理毒理

阿戈美拉汀是一种褪黑素受体激动剂和5-HT 2C受体拮抗剂。动物研究结果显示，阿戈美拉汀能校正昼夜节律紊乱动物模型的昼夜节律，使节律得以重建。阿戈美拉汀在多种抑郁症动物模型中显示出抗抑郁作用。 阿戈美拉汀能特异性地增加前额皮质去甲肾上腺素和多巴胺的释放，细胞外5-羟色胺水平未见明显影响。受体结合试验结果显示，阿戈美拉汀对单胺再摄取无明显影响，对α、β肾上腺素受体、组胺受体、胆碱能受体、多巴胺受体以及苯二氮卓类受体无明显亲和力。

遗传毒性：阿戈美拉汀Ames试验、小鼠淋巴瘤细胞基因突变试验、人外周血淋巴细胞遗传学试验、程序外DNA合成（UDS）试验、微核试验结果均为阴性。

生殖毒性：阿戈美拉汀在经口给药剂量低于240mg/kg时对雌性和雄性大鼠的生育力未见影响（NOAEL），在此剂量下动物暴露量比人用剂量25mg时暴露量高300倍。胚胎胎仔发育毒性试验中，大鼠中阿戈美拉汀剂量高至640mg/kg时未见胚胎毒性和致畸性；家兔剂量高至450mg/kg时未见胚胎毒性和致畸性；上述剂量母体暴露量大约分别是人用剂量25mg时物暴露量的720和300倍。大鼠围产期毒性试验中，在母体暴露量为人用剂量25mg时暴露量的280倍时，未见明显异常。

致癌性：大鼠与小鼠分别经口给予维度新40、120、360mg/kg或125、500、2000mg/kg，连续104周，按暴露量计算分别相当于人用剂量25mg时暴露量的7.5、22、110倍或5、10、47.5倍。大鼠中，剂量≥120mg/kg的雄性动物肝腺瘤发生率增加，360mg/kg剂量组雄性动物肝癌发生率增加；小鼠中，500、2000mg/kg剂量组肝腺瘤发生率增加，2000mg剂量下雌雄动物的肝癌的发生率增加。

## 1.2．药代动力学

吸收和生物利用度：阿戈美拉汀口服后吸收快速且良好（≥80%）。绝对生物利用度低（口服治疗剂量<5%），个体间差异较大。与男性相比，女性的生物利用度较高。口服避孕药会增加药物的生物利用度，而吸烟会使生物利用度降低。服药后1-2小时内达到血浆峰浓度。在治疗剂量范围内，阿戈美拉汀的系统暴露随剂量升高而成比例地增加。高剂量时，首过效应达到饱和。进食（标准饮食或高脂饮食）不影响阿戈美拉汀的生物利用度或吸收率。高脂饮食会增加个体差异。

分布：稳态分布容积约为35L，血浆蛋白结合率为95%，与药物血浆浓度无关，不受个体年龄或者肾脏功能的影响。但肝功能损害患者游离药物浓度可升高1倍。

生物转化：阿戈美拉汀口服后主要经肝脏CYP1A2同工酶迅速代谢，CYP2C9和CYP2C19同工酶也参与阿戈美拉汀的代谢，但作用较小。主要代谢产物羟化阿戈美拉汀和去甲基阿戈美拉汀均无活性且在体内迅速结合，并经尿液排出。

消除：阿戈美拉汀消除速率快，平均的血浆消除半衰期为1-2小时，清除率较高（约为1100mL/min），主要以代谢产物的形式经尿液排泄，其中原型药物成分可忽略不计。重复给药不会改变药物的药代动力学过程。

## 1.3．不良反应和副作用

常见的有头疼、头晕、嗜睡、失眠、偏头痛；恶心、腹泻、便秘、上腹部疼痛、多汗、背痛、视觉疲劳等。对驾驶和操作机械能力可能有影响。

## 1.4．临床应用

用于治疗成人抑郁症。中度或重度肾功能损害患者慎用。肝病患者禁用，儿童及18岁以下青少年患者不推荐使用。

## 2．试验目的

## 其目的是研究中国健康男性志愿者口服法国施维雅公司生产的阿戈美拉汀片（商品名：维度新，25mg/片）的体内变异系数，为评价以施维雅公司参比制剂与国内厂家生产的试验制剂间的生物等效性提供参考。

## 3．研究方案依据

本临床研究方案主要依照《赫尔辛基宣言》、《中华人民共和国药品管理法》、《药品注册管理办法》、《药物临床试验质量管理规范》、《化学药物制剂人体生物利用度和生物等效性研究技术指导原则》、《药物Ⅰ期临床试验管理指导原则（试行）》、《药物临床试验生物样本分析实验室管理指南（试行）》有关规定制定。

## 4．试验负责人及主要研究人员简介

阳国平，临床研究机构主任，教授，临床药理学博士，经过国家GCP培训。

叶 玲，临床监护医生，临床医学学士，经过国家GCP培训。

阳晓燕，临床监护医生，临床医学学士，经过国家GCP培训。

裴 奇，分析测试，药学硕士，经过国家GCP培训。

谭鸿毅，分析测试，药学学士，经过国家GCP培训。

杨 双，研究护士，护理学大专，经过国家GCP培训。

李兰倪，研究护士，护理学大专，经过国家GCP培训。

姚 安，研究护士，护理学大专，经过国家GCP培训。

华 烨，质控，药学学士，经过国家GCP培训。

王 艳，项目助理，药学硕士在读，经过国家GCP培训

## 5．试验用药

药名：阿戈美拉汀片

商品名：维度新

来源：法国施维雅

批号：893158

规格：25mg /片；

有效期至：2014年7月

储存条件：密闭，25℃以下保存。

## 6．受试者选择

## 6.1．筛选步骤

本研究的对象是年龄18~40岁健康志愿者并在试验前1周内按以下程序进行受试者的筛选：

1. 人口学统计资料；
2. 生命体征；
3. 询问病史，近期和当前的合并用药史；
4. 心电图；
5. 临床实验室检查（具体检查项目见表1，在试验前1周内检查）；
6. 评价入选／排除标准；
7. 符合要求的志愿者按报名先后入组，并随机分组。

### 6.2． 入选标准

符合下列所有条件的受试者进入本试验

1. 性别：男性；
2. 年龄：18-40岁之间，同批年龄相差不超过10岁；
3. 体重：体重大于50 kg，受试者体重指数（BMI）在19-24 kg/m^2^之间（BMI=体重（kg）/身高^2^（m^2^））；
4. 依据体格检查、病史、生命体征、心电图和临床实验室检查的结果，研究者认为受试者的健康状况良好，无心、肝、肾、消化道、神经系统、精神异常及代谢异常等病史；经全面体格检查显示心电图、血压、心率、呼吸状况以及实验室检查包括血、尿常规，肝、肾功能等各项生化检查均无异常或异常无临床意义（以临床医师判断为准）；
5. 受试者无过敏史，试验前两周内未服用任何其他药物；
6. 受试者无吸烟、饮酒嗜好；
7. 受试者必须在试验前对本研究知情同意，并自愿签署了书面的知情同意书；

### 6.3．排除标准

符合一条或多条下列标准的受试者将被排除：

1. 过敏体质（对两种以上药物、食物、花粉过敏）；或已知对本药组分有过敏者；有明确的食物等过敏史者；
2. 体位性低血压史者；
3. 有特异性变态反应病史（哮喘、风疹、湿疹性皮炎）、心律失常、支气管和心血管疾病史，糖尿病，甲状腺功能亢进，帕金森综合症，或者能够干扰试验结果的任何其他疾病或生理情况；
4. 正在或曾经接受过胃肠道问题、痉挛、消化道溃疡、尿路梗塞、机械性肠梗阻、输尿管痉挛、胆道疾病、抑郁障碍或肝脏疾病治疗,或者其它能影响药物吸收和代谢的疾病；
5. 已知的能够影响静脉取血的严重出血因素；
6. 在过去五年内有药品滥用史或者阳性尿药检测结果；
7. 试验前及试验期间心电图异常（具有临床意义）或生命体征异常者（收缩压<90 mmHg或>140 mmHg，舒张压<50 mmHg或>90 mmHg；心率<50 bpm或>100 bpm）；
8. HIV,乙肝表面抗原（HbsAg）或丙肝病毒（HCV）检测阳性者；
9. 试验前6个月内或试验期间经常饮酒者，即每周饮酒超过14单位酒精（1单位=360 mL啤酒或45 mL酒精量为40%的烈酒或150 mL葡萄酒）；
10. 试验前3个月及试验期间服用软毒品（如：大麻）或试验前一年及试验期间服用硬毒品（如：可卡因、苯环己哌啶等）者；
11. 试验前30天或试验期间使用过任何抑制或诱导肝脏对药物代谢的药物（如：诱导剂—巴比妥类、卡马西平、苯妥英、糖皮质激素、奥美拉唑；抑制剂—SSRI类抗抑郁药、西咪替丁、地尔硫卓、大环内酯类、硝基咪唑类、镇静催眠药、维拉帕米、氟喹诺酮类、抗组胺类）者；
12. 在试验前14天内服用过任何药物者；
13. 试验前30天内服用了任何临床试验药物或参加了任何药物临床试验者；
14. 试验前三个月献血或作为受试者被采样者；
15. 患有凝血功能障碍或血栓栓塞性疾病者；
16. 试验前3个月每日吸烟量多于1支，或试验期间使用过任何烟草类产品者；
17. 任何食物过敏或对饮食有特殊要求，不能遵守统一饮食者；
18. 有恶性肿瘤病史者；
19. 有严重的偏头痛或头痛病史者；每天饮用过量茶、咖啡和/或含咖啡因的饮料（8杯以上）者；
20. 研究者认为不应纳入者。

### 6.4． 剔除标准：

根据GCP及ICH指导原则，所有受试者在试验期间都有权利在研究任何时期退出试验，此外，试验过程中申办方、研究者或其授权人员有权剔除符合下列情况的受试者。

1. 继续参与临床试验会对受试者造成不必要的伤害
2. 受试者依从性差，不能按时按量用药；
3. 使用其他影响耐受性判断或影响药动学结果的药物或食物；
4. 同时使用了其他治疗以致影响评价；
5. 试验药物的使用与临床试验方案不符；
6. 药动学试验中采血后血样处理、保存、运输方法失误；
7. 参与试验后发现不符合入组标准的受试者；
8. 参与试验后发现符合排除标准的受试者；
9. 脱落的受试者；
10. 除上述情况外，试验过程中其他偏离或违反方案，并对药物等效性评价或安全性评价造成影响的受试者。

**表1 受试者观察指标的具体要求**

| **项目** | **要求及观测指标** |
| --- | --- |
| 人口学资料 | 年龄、性别、民族、身高、体重、体重指数 |
| 生命体征 | 体温、血压、心率、呼吸 |
| 心电图 | 标准的12导联ECG |
| 血常规 | 白细胞计数、红细胞计数、血红蛋白、血小板计数、中性粒细胞百分比 |
| 尿常规 | 蛋白质、葡萄糖、酮体、白细胞、潜血、pH值 |
| 肝肾功能 | 丙氨酸氨基转移酶、天门冬氨酸氨基转移酶、总蛋白、白蛋白、总胆红素、直接胆红素、肌酐、尿素氮、尿酸 |
| 血糖 | 空腹血糖 |
| 凝血功能 | 凝血酶原时间（PT）、活化部分凝血活酶时间（APTT） |
| 输血四项 | 乙肝表面抗原(HBsAg)、丙肝病毒抗体(HCV-AB)、梅毒血清反应素(RPR)、人类免疫缺陷病毒抗体（Anti-HIV） |

## 7. 剂量及确定依据

按照化学药物制剂人体生物利用度和生物等效性研究技术指导原则，以及阿戈美拉汀片说明书和该药物的临床应用情况，设定给药剂量为25mg。

### 8. 试验设计及方案描述

### 8.1．受试者例数

预试验16例。根据预试验的结果和统计学的要求来决定正式试验的受试者例数。

### 8.2．给药途径和给药方案

采用口服的方式给药，用温开水送服，需整片吞服，不得分割、压碎或咀嚼。

给药前禁食10小时以上，于第二天早上单次口服阿戈美拉汀片（25 mg）或安慰剂。服药前及服药后2小时内控制饮水，服药后4小时、10小时进食标准午餐和晚餐（统一的低脂清淡饮食），剩余3周期按照试验安排（见随机分组表）空腹服用阿戈美拉汀片或安慰剂中的一种，250 mL温水送服。周期间的洗脱期为7天。

### 8.3试验设计

2012年6月26日国家新药评审中心（CDE）发布的《开展阿戈美拉汀口服制剂人体生物等效性试验时应关注的问题》指出，阿戈美拉汀口服制剂绝对生物利用度很低，约为3-4%，且变异很大。阿戈美拉汀不同试验口服给药后药物暴露AUC变异系数（CV）约为100-150%。个体间变异是主要来源，但个体内变异也非常大，其绝对生物利用度的个体间和个体内变异系数分别为157%和104%。一般认为，以AUC和Cmax计算的个体内变异系数大于或等于30%的药物即属于高变异药物，采用生物等效性研究通常的18-24例受试者，会由于样本例数少而无法获得足够的把握度，从而增加试验失败的风险。尽管对于高变异药物的生物等效性评价，除增加例数外还可采用重复交叉设计等方法，但增加受试者例数仍然是最简单和有效的方法。目前还没有阿戈美拉汀AUC和Cmax个体内变异系数的公开数据报道，这种情况下，可以参照欧盟关于生物等效性研究的指导原则，先采用参比制剂进行一个小规模的重复交叉试验（至少12例），以获得该药物准确可靠的个体内变异系数，然后根据该数据进行受试者例数的估算。

基于上述原因，本试验在正式试验前增设预试验流程。采用四周期交叉试验设计，随机入选16名健康男性志愿受试者，随机分成4组，每组4人，进行参比制剂的单次给药生物等效性的预试验，周期间的清洗期为1周，预试验服药顺序见表2。主要目的是获得该药物准确可靠的个体内变异系数，然后根据该数据进行正式试验受试者例数的估算。另外也了解预先设计的采血时点、给药剂量是否恰当。

**表2 预试验随机分组表**

| **体重顺序号** | **区组** | **随机数** | **分组** |  | **按随机号排序** | |  |  |  |
| --- | --- | --- | --- | --- | --- | --- | --- | --- | --- |
|  |  |  |  |  | **随机数** | **体重号** | **分组** | **试验号** | **给药方案** |
| 1 | 1 | 0.86 | 1 |  | 0.57 | 11 | 1 | 1 | P-R-R-R |
| 2 | 1 | 3.08 | 3 |  | 0.59 | 9 | 2 | 2 | R-P-R-R |
| 3 | 1 | 1.49 | 2 |  | 0.86 | 1 | 1 | 3 | P-R-R-R |
| 4 | 1 | 8.43 | 4 |  | 1.49 | 3 | 2 | 4 | R-P-R-R |
| 5 | 2 | 15.26 | 4 |  | 2.17 | 10 | 3 | 5 | R-R-P-R |
| 6 | 2 | 13.42 | 3 |  | 2.32 | 13 | 1 | 6 | P-R-R-R |
| 7 | 2 | 8.81 | 1 |  | 3.08 | 2 | 3 | 7 | R-R-P-R |
| 8 | 2 | 11.26 | 2 |  | 5.02 | 14 | 2 | 8 | R-P-R-R |
| 9 | 3 | 0.59 | 2 |  | 6.89 | 15 | 3 | 9 | R-R-P-R |
| 10 | 3 | 2.17 | 3 |  | 8.43 | 4 | 4 | 10 | R-R-R-P |
| 11 | 3 | 0.57 | 1 |  | 8.81 | 7 | 1 | 11 | P-R-R-R |
| 12 | 3 | 10.51 | 4 |  | 10.16 | 16 | 4 | 12 | R-R-R-P |
| 13 | 4 | 2.32 | 1 |  | 10.51 | 12 | 4 | 13 | R-R-R-P |
| 14 | 4 | 5.02 | 2 |  | 11.26 | 8 | 2 | 14 | R-P-R-R |
| 15 | 4 | 6.89 | 3 |  | 13.42 | 6 | 3 | 15 | R-R-P-R |
| 16 | 4 | 10.16 | 4 |  | 15.26 | 5 | 4 | 16 | R-R-R-P |

注：SPSS 13.0； 种子号：20120828； R为参比制剂；P为安慰剂

如果试验过程中受试者中途退出试验，而非终止试验，且受试者总例数少于12例，需要选择体检合格的替补受试者完成试验。替补受试者的号码为退出受试者的号码加100。如5号退出则其替补号码为5+100=105。若替补受试者中途也退出试验，则入选第二替补受试者，其号码为第一替补者号码加100，如上例为105+100=205。替补受试者接受退出受试者相同编号的试验药物。

### 8.4．试验方法

**第一周期**：受试者于试验前1天晚上9点入住Ⅰ期病房，给药前禁食10小时以上，于试验当天早上单次口阿戈美拉汀片(25 mg)或安慰剂，250mL温开水送服。服药前及服药后2小时内控制饮水，服药后4小时、10小时进食标准午餐和晚餐（统一的低脂清淡饮食）。于服给药前（0h）及给药后15min、30min、45 min、1.0h、1.5h、2.0h、2.5h、3.0h、4.0h、5.0h、6.0h、8.0h、10.0h、12.0h、16.0h采集静脉血5ml用于药物浓度分析。试验过程记录不良事件。血样采集后置于事先已经贴好标签的肝素抗凝试管内，1小时内离心（3000 rpm，10 min）分离血浆，血浆转移至EP管中置−20℃冰箱保存，待测。

洗脱期**：**7天。

**第二、三、四周期**：受试者均按研究方案分别服用阿戈美拉汀片(25 mg)或安慰剂，重复第一周期程序。

### 8.5.试验前后体检及试验期间随访观察

健康受试者在筛选期接受全面的检查，包括人口统计学资料、生命体征（血压、心率、体温、呼吸）、心电图、血常规、尿常规、肝肾功能、血糖、输血四项、凝血功能等检查（具体项目见表1）。

试验期间受试者需要于每周期给药开始前（0h）、给药结束后2h、12h、24h接受生命体征检查，询问并记录不良事件及合并用药情况。

在试验期间（第1天给药前的1h~试验结束时）发生自觉症状时，要求受试者向试验负责医生汇报，并由试验负责医师确认后记录在原始病历中。

在试验结束时受试者接受出组身体检查，包括生命体征（血压、心率、体温、呼吸）、心电图、血常规、尿常规、肝肾功能、血糖等项目的检查。

如出现有临床意义的异常，继续随访至转归。

具体安排见试验流程表。

### 8.6． 生物样品分析方法

根据相关文献报道，拟采用HPLC-MS/MS法检测阿戈美拉汀的血浆药物浓度。

### 9．数据处理与统计

### 9.1. 数据提供

提供所有受试者各个时间点参比制剂的药物浓度测定数据，提供每个受试者的血药浓度-时间曲线（C-T曲线）和平均C-T曲线以及C-T曲线各个时间点的标准差。不能随意剔除任何数据。数据经核对无误后，双份录入计算机数据库。

### 9.2．药动学参数估算方法

采用非房室模型求算药动学参数，药动学分析应包括以下药动学参数

1. Cmax 峰浓度，为实测值
2. Tmax 达峰时间，为实测值
3. AUC0-t 0到t时间血药浓度-时间曲线下面积，采用梯形法计算：

AUC0-t =∑(Ci+Ci-1)(ti-ti-1)/2

1. λz 表观末端消除速率常数，由消除相浓度点取半对数线性回归而得
2. t1/2 表观末端消除半衰期，按下式计算：t1/2=0.693/λz
3. AUC0-∞ 0时到无穷时的血药浓度-时间曲线下面积AUC0-∞=AUC0-t + Ct/λz (Ct为最后一个可测得时间点的血药浓度)

### 10．统计分析

根据阿戈美拉汀自身对照重复试验结果，对获得经对数转化的主要药动学参数AUC和Cmax进行多因素方差分析，包括给药周期、给药顺序及嵌套在给药顺序内的受试者个体内因素，假定其中得到的残差全部由受试者个体内变异构成，则可根据公式求得药物在个体内差异（即变异系数，CV）。

### 11. 安全性评价

安全性评价不是本研究的重点，但为最大限度的保护受试者的安全和权益并考察试验药物的安全性，试验过程中由临床医师进行监护，注意观察和询问受试者的各种反应，及时记录不良事件的性质、发生频率、处置及转归，并进行相关性分析，同时对试验前后实验室检查结果进行分析。

### 11.1 医疗监护

研究方案必须通过中南大学湘雅三医院伦理委员会批准后方可进行，本试验全过程由湘雅三医院医生、护士做好不良反应的监护。监护医生在服药后前4小时不应离开Ⅰ期病房区域，且在整个试验的所有其它时间里可以迅速与医生取得联系。监护期内随时观察受试者一般不良反应，对于出现的不良反应，应及时进行对症治疗，监护期外需进行随访，以确定是否存在其他不良反应。

对所有不良反应事件，监护医生将追踪并取得足够的信息，以确定此不良事件的原因（如有研究药物本身或其他原因引起），监护医生及上级主管医生会同研究者、项目负责人评估不良事件产生的原因并由监护医生在临床采样记录中写明这一评估。如果不良事件或其后遗症持续存在，即使在研究药物停用以后也需随访此不良事件。这种随访将持续到此不良事件或其后遗症消除。

在临床试验过程中发生的所有严重不良事件，不论是否怀疑与研究药物有关，监护医生将立即对受试者采取适当的保护措施，在24小时内由研究者电话报告监查员，同时书面报告国家食品药品监督管理局、伦理委员会以及申办单位，并在报告上签名并注明时间。

### 11.2不良事件

不良事件是病人或临床试验受试者接受一种药品后出现的不良医学事件，但并不一定与治疗有因果关系。

所有不良事件的信息，不论是受试者自述的、研究者问出的、或通过体检、实验室检查、或其它方法发现的，均应收集并记录在不良事件病例报告表上，并适当随诊。不良事件是使用研究药物后发生的任何非预期出现的体征、症状或医学情况，即使它们与研究药物无关。

研究开始前已存在的医学情况/疾病，只有在研究开始后恶化，才算是不良事件。研究开始前、但在签署知情同意书之后发生的不良事件应记录在观察表上。异常实验室化验值或检查结果只有当它们引起临床症状或体征、或需治疗、或认为有临床意义才构成不良事件，这种情况应在不良事件病例报告表上记录症状、体征、或与其有关的诊断。

不良事件的严重程度是对研究者确定的或受试者报告的不良事件的范围或强度的定性评价。严重程度不反映事件在临床上的严重性，而只描述痛苦或发生的程度或范围，也不反映与试验药物的相关性。

**不良事件严重程度判定标准：**

轻度：仅受试者认为是明显症状，不影响行为和功能，不需用处方药来缓解症状，但可根据受试者的要求给予处方药。

中度：出现明显症状使受试者感到不适，每天的行为活动受到影响，受试者可以继续进行试验，可能需要对症治疗。

重度：引起严重不适症状，受试者不能继续参加该试验，症状程度可能造成终止试验药物的治疗，可进行对症治疗和/或要求受试者住院治疗。

**不良事件与试验药物相关性的判定标准**（SFDA：《药品不良反应报告与监测工作手册》，2005年）：

药物与不良事件间的关系被判断为：肯定有关、很可能有关、可能有关、可能无关、不可能有关5级。评价方法见下表。

表3 **不良事件与试验药物相关性的判定标准**

|  | ① | ② | ③ | ④ | ⑤ |
| --- | --- | --- | --- | --- | --- |
| 肯定有关 | ＋ | ＋ | ＋ | ＋ | － |
| 很可能有关 | ＋ | ＋ | ＋ | ？ | － |
| 可能有关 | ＋ | ＋ | ± | ？ | ± |
| 可能无关 | ＋ | － | ± | ？ | ± |
| 不可能有关 | － | － | － | － | ＋ |

注：＋表示肯定； －表示否定； ±表示难以肯定或否定； ？表示情况不明

①用药与不良反应/事件的出现有无合理的时间关系？

②反应是否符合该药已知的不良反应类型？

③停药或减量后，反应是否消失或减轻？

④再次使用可疑药品是否再次出现同样反应/事件？

⑤反应/事件是否可用并用药的作用、患者病情的进展、其他治疗的影响来解释？

### 11.3．严重不良事件

严重不良事件是临床试验过程中发生需住院治疗、延长住院时间、伤残、影响工作能力、危及生命或死亡、导致先天畸形等事件。

所有在临床研究中出现的严重不良事件都应在研究人员知晓后的24小时内报告伦理委员会、国家食品药品监督管理局及申办者。相关的联系方式在单独的文件中提供。

严重不良事件的内容应填写在SAE报告表内以传真形式报给申办者。报告必须有研究医生的签名。如果在第一次报告SAE时无法填写或传真SAE报告表，可以通过电话报告，但需在电话报告后的1个工作日内由研究医生填好SAE报告表传真至公司。

在研究结束或受试者提前退出时没有解决的严重不良事件都必须随访直至达到下列任何情况之一：

事件解决

事件稳定

事件返回基线水平，如果有基线值可提供

事件可被归因于研究药物之外的药物或者与研究行为无关的因素，或者当更多的信息不太可能再获得时（病人或医护人员拒绝提供更多的信息，或有证据说明已尽最大的努力后病人仍然失访）。

### 11.4．阿戈美拉汀片可能出现的不良反应及抢救措施

可能出现的不良反应有头晕(9.3%)、鼻塞(4.8%)、失眠(1.6%)、头痛、恶心、疲倦、口干、腹泻、嗜睡、便秘、腹痛、多汗、背痛、视觉疲劳等

**治疗**：若发生不可耐受的不良反应立即停药并对症治疗。

**抢救药品：**0.1%肾上腺素、多巴胺、阿拉明、阿托品、利多卡因、地塞米松、尼可刹米等。

**抢救设备：**吸氧装置、吸引器、气管插管、气囊心电监护仪、除颤器等。

### 11.5．终止试验标准

1. 在试验过程中出现了严重不良反应（危及生命，或影响正常的一般性工作和生活），应终止试验。
2. 如半数以上受试者出现同一重度不良反应，应终止试验
3. 试验期间任何受试者出现严重不良事件，则先中止研究，由研究者和申办方共同决定是否终止本研究。

### 12． 伦理要求

在开始进行该试验之前，试验方案、推荐的知情同意书以及其它给受试者的信息必须经中南大学湘雅三医院医学伦理委员会审查。对试验方案所做的任何修改必须再经伦理委员会批准或备案。

研究者必须向每一位受试者解释该试验的性质、目的、有关程序、预期时间、潜在风险和包括的利益、以及可能出现的任何不适。每位受试者必须知道参加该试验是自愿的，他在任何时候都可以退出该试验以及撤回知情同意，不会影响他随后的治疗或与治疗医生的关系。

该知情同意书应采用标准的书写格式给出，并使用非专业语言。受试者在签字和注明日期之前应阅读和考虑其声明，并应获得签署文件的复印件。未获得知情同意，受试者不能进入该试验。

### 13 . 试验管理与质量控制

### 13.1．管理机构和实施GCP的情况

1. 申办者和研究者均应采用标准操作程序执行临床试验的质量控制和质量保证系统。
2. 原始资料必须符合中国GCP要求。
3. 实验室检查结果必须准确可靠。
4. 所用观察结果和发现都应加以核实，以保证数据的可靠性。
5. 建立完整的试验组织机构，明确各级人员职责。
6. 由主要研究者负责全面质量控制或委托质控员进行全程质控，执行各级人员职责。
7. 由主要研究者负责设计研究方案、临床采样记录，并经申办者同意后使用。试验结束后写出试验的总结报告。
8. 由指定研究者负责制定试验实施细则和SOP，在试验中使用。
9. 试验前由试验小组组织全体参加者学习方案，试验参加人员均经过GCP培训并获相应证书。
10. 参加试验的医师、护士要严格遵守方案的规定，按程序进行，不得随意更改。
11. 由指定统计人员负责数据的全面统计学处理。
12. 由专人负责临床采样记录的保管，试验结束后统一上交机构办公室归档。

### 13.2．各方职责

申办方向试验中心提供试验相关的研究者手册等资料和试验经费，免费提供经检验合格的试验药物（包括对照药物及对照品）、试验材料（包括：药物临床试验批件，申办方营业执照、药品生产许可证、GMP证的复印件，药品检验报告书原件），申办方与研究者共同制定试验方案并经双方确定。研究者根据试验方案，按照国家现行的法规进行试验。

试验中心应对申办方提供的全部信息严格保密，同时也要求其它试验参与人员和伦理委员会采取同样的保密措施。提供给试验中心的信息资料未得到申办方的书面许可不得泄漏给他人。

试验的全部资料和试验结果归研究方和申办方共同所有。研究者没有得到申办方的同意不得自行发表。任何希望发表的文章都应在送出前提交一份给申办方，申办方将复审其准确性（以免与递送SFDA的报告不一致），确认保密信息没有泄漏并补充相关信息。

### 13.3．方案修订

方案的修订：本方案经伦理委员会批准后，若要修改，需制定“方案修改说明书”，并由主要研究者签字，经申办者同意方可修改方案。方案修改后，需报伦理委员会批准或备案后方可执行。

任何参加试验的人员不得违背方案。若发生违背方案事件，需写出说明并通知申办者，后者有权决定试验是否继续或提出处理方案。

### 13.4．受试者管理

受试者于试验前一天晚上9时前进入I 期临床病房，开始禁食，并且不得自带任何食品，可以饮水。早上6:00开始不得随意饮水，早上8:00开始给药至给药后2小时内控制饮水。以后不做规定。服药后4小时、10小时进食标准午餐和晚餐（统一的低脂清淡饮食），第二、三、四周期与第一周期相同。

在研究前24小时和研究期间，禁止饮用含酒精和咖啡因的饮料，并且不能进行剧烈运动。

试验期间不得离开I 期临床病房。

洗脱期应与受试者保持联系，不得服用试验方案中禁止的药物，不要进行剧烈运动，预防感冒。

### 13.5．试验药物管理

试验药物按照说明书的要求在适当的条件下保存，药物保存必须安全带锁，由研究者负责保管。试验药物的运送、接受和分发必须由中南大学湘雅三医院临床机构被授权的人员进行登记，且必须有分发给每位受试者试验药物的日期和数量的准确记录，以备随时检查。所有药物仅用于该试验方案，不能用于其它目的。研究者不能销毁任何药物标签或剩余的或未使用的药物。未使用的药物在完成试验后退还给申办者。

### 13.6．给药依从性管理

研究者当场给受试者服药，并有人核对，以保证所有受试者100％的服药依从性，并将具体给药时间记录在相应的表格中。

### 13.7．受试者退出试验的管理

受试者可以不以任何理由退出试验。加强管理，严格按照试验方案进行试验，尽量避免非安全原因导致的受试者退出。受试者退出后应详细记录退出原因，并进行替代。替代受试者必须重复他要替代的那个受试者相同的试验。

### 13.8．伴随治疗

在整个试验过程中，必要时，研究者可以根据情况批准受试者使用其它药物，如使用其它药物，应详细记录用药情况和原因。除此以外，受试者不允许服用试验药物以外的任何其它药物。

### 13.9．数据记录与文件保留

临床采样记录上有关受试者数据应以代码方式记录，受试者只能通过受试者号码或其名字首字母识别。

研究方案所要求的所有信息必须提供，任何省略要给出理由。监查员要检查记录的完整性、准确性和字迹清晰，研究者是否签字。

临床采样记录的所有记录必须用黑色签字笔清晰填写，以确保复印页的清晰易读。不正确的记录必须在其上划一条横线来进行修改，以便原记录仍然可见，然后在旁边做修改记录。修改记录必须由研究者签字和注明日期。

研究者必须保留参加试验的每位受试者的原始文件。临床采样记录上的所有信息必须来源于这些原始文件，原始文件应含有所有人口统计学信息和治疗信息，包括实验室检查数据、心电图等，也包括指明试验编号和试验题目的知情同意书签字件。

下列基本文件必须由研究者保留至国家或国际法规所要求的时间（通常为临床研究结束后或最终上市批准后5年时间），申办方应通知研究机构相关试验记录不再需要的时间。基本文件包括：

1. 国家食品药品食品监督管理局临床研究批件；
2. 试验药品质检报告书；
3. 伦理委员会对研究方案和所有修改的批准件；
4. 所有原始文件和实验室检查记录；
5. 临床采样记录；
6. 受试者知情同意书；
7. 全部色谱分析方法学及受试者血药浓度分析测定图谱；
8. 药动学参数计算结果；
9. 研究原始记录本；
10. 其它相关的试验文件。

### 13.10．个人资料的保密

本研究仅收集和处理那些局限于对药物的有效性、安全性、质量和应用等进行研究时所需的受试者的数据。

在收集和使用这些数据时会充分确保其保密性并且遵守相关的保护受试者隐私的法律和法规。

### 13.11．质量控制

试验启动前必须对所有研究者进行试验方案培训，试验过程中严格执行各项SOP，项目负责人应认真审核各项记录，核对各项数据，申办方应派合格监查员对试验过程进行监督，对试验数据进行核对。

### 14．参考文献

[1].胡茂荣, 陈晋东, 李乐华, 国效峰, 陆晓姿. 阿戈美拉汀: 一种新型抗抑郁药。5 应0000000000000000000000000000000000000000000000000000000000000000000000000000000000000000000000000000000000000000000000000000. 中国新药与临床杂志, 2009, 28(2): 81-85.

[2]. [Patil SR](http://www.ncbi.nlm.nih.gov/pubmed?term=Patil SR%5BAuthor%5D&cauthor=true&cauthor_uid=22282085), [Nerurkar KK](http://www.ncbi.nlm.nih.gov/pubmed?term=Nerurkar KK%5BAuthor%5D&cauthor=true&cauthor_uid=22282085), [Kalamkar AM](http://www.ncbi.nlm.nih.gov/pubmed?term=Kalamkar AM%5BAuthor%5D&cauthor=true&cauthor_uid=22282085), [Pukale V](http://www.ncbi.nlm.nih.gov/pubmed?term=Pukale V%5BAuthor%5D&cauthor=true&cauthor_uid=22282085), [Mangaonkar KV](http://www.ncbi.nlm.nih.gov/pubmed?term=Mangaonkar KV%5BAuthor%5D&cauthor=true&cauthor_uid=22282085), [Pingale SG](http://www.ncbi.nlm.nih.gov/pubmed?term=Pingale SG%5BAuthor%5D&cauthor=true&cauthor_uid=22282085). Validated LC-MS/MS method for quantification of agomelatine in human plasma and its application in a pharmacokinetic study. [J Mass Spectrom.](http://www.ncbi.nlm.nih.gov/pubmed?term=Validated LC–MS%2FMS method for quantification of agomelatine in human plasma and its" \l "%23) 2012 Jan;47(1):23-8.

[3]. 阿戈美拉汀片（维度新）说明书

[4]. 赵德恒. 开展阿戈美拉汀口服制剂人体生物等效性试验时应关注的问题. <http://www.cde.org.cn/dzkw.do?method=largePage&id=312714>
